# Reply to M. Dhanushkodi

**DOI:** 10.1200/JGO.2016.003517

**Published:** 2016-05-18

**Authors:** Atlal Abusanad

**Affiliations:** King Abdulaziz University, Jeddah, Saudi Arabia

In a letter to the editor in response to our recent article in *Journal of Global Oncology* titled “Outcomes of Saudi Arabian Patients With Nasopharyngeal Cancer Treated With Primarily Neoadjuvant Chemotherapy Followed by Concurrent Chemoradiotherapy,”^1^ Dhanushkodi^2^ raises some interesting points. The Meta-Analysis of Chemotherapy in Nasopharynx Carcinoma (MAC-NPC) demonstrated improved overall survival (OS) by adding concomitant chemotherapy to radiotherapy (RT) in patients with NPC, but the potential effect of neoadjuvant chemotherapy (NACT) and adjuvant chemotherapy on OS was not established. Six trials comparing RT plus concomitant chemotherapy with the same RT with or without concomitant chemotherapy plus induction (NACT) chemotherapy were included in this meta-analysis. There was a statistically significant improvement in progression-free survival at 5 years (47% *v* 39%; hazard ratio, 0.81; 95% CI, 0.69 to 0.95) but 5-year OS was not significant (57% *v* 55%; hazard ratio, 0.96; 95% CI, 0.80 to 1.16) with induction chemotherapy.^3^ From our experience, NACT was useful for prompt symptom relief from large primary tumors (T4 lesions) or advanced nodal disease, or when delivery of a full course of RT was not possible because of critical surrounding structures, which was the case in the majority of our patients. Nonetheless, the meta-analysis did not examine such a potential beneficial effect of NACT on symptom relief and RT planning and delivery. Hence, the notion of no benefit of such an approach is not correct. We used induction TPF with docetaxel 75mg/m^2^ on day 1, cisplatin 75 mg/m^2^ on day 1, and 5-FU 750 mg/m^2^/day for 5 days (1→5).

Palliative RT to the primary tumor was given to four patients (10%); two patients had metastatic disease and two patients had a poor performance status, which prevented the administration of systemic chemotherapy.

As indicated in our review, selecting patients for NACT was based on the treating medical and radiation oncologists’ preferences, but overall, patients with nodal disease received NACT regardless of the tumor extent.^1^ Likewise, and because of the retrospective nature of this review, the choice between once per week and every 3 weeks cisplatin was at the treating physician’s discretion. No patient underwent resection of residual cervical lymphadenopathy. Molecular testing for Epstein-Barr virus markers on archived tumor samples is underway. The data on managing subsequent relapses and the possibility of a tobacco and alcohol association with NPC are beyond the scope of this review.

## References

[1] Al-Wassia R, Abusanad A, Awad N, et al: Outcomes of Saudi Arabian patients with nasopharyngeal cancer treated with primarily neoadjuvant chemotherapy followed by concurrent chemoradiotherapy. J Glob Oncol 2:123-128, 2016 10.1200/JGO.2015.001743 PMC5495452 28717691

[2] Dhanushkodi M: Nasopharyngeal cancer outcomes from Saudi Arabia. J Glob Oncol 2:164, 2016 10.1200/JGO.2016.003418 PMC5495456 28717696

[3] Blanchard P, Lee A, Marguet S (2015). Chemotherapy and radiotherapy in nasopharyngeal carcinoma: An update of the MAC-NPC meta-analysis. Lancet Oncol.

